# Hydrogen Sulfide Sensitizes *Acinetobacter baumannii* to Killing by Antibiotics

**DOI:** 10.3389/fmicb.2020.01875

**Published:** 2020-08-07

**Authors:** Say Yong Ng, Kai Xun Ong, Smitha Thamarath Surendran, Ameya Sinha, Joey Jia Hui Lai, Jacqueline Chen, Jiaqi Liang, Leona Kwan Sing Tay, Liang Cui, Hooi Linn Loo, Peiying Ho, Jongyoon Han, Wilfried Moreira

**Affiliations:** ^1^Antimicrobial Resistance Interdisciplinary Research Group (AMR IRG), Singapore-MIT Alliance for Research and Technology (SMART) Centre, Singapore, Singapore; ^2^Critical Analytics for Manufacturing Personalized-Medicine Interdisciplinary Research Group (CAMP IRG), Singapore-MIT Alliance for Research and Technology (SMART) Centre, Singapore, Singapore; ^3^Department of Biological Engineering, Massachusetts Institute of Technology, Cambridge, MA, United States; ^4^School of Biological Sciences, Nanyang Technological University, Singapore, Singapore; ^5^Department of Electrical Engineering and Computer Science, Massachusetts Institute of Technology, Cambridge, MA, United States

**Keywords:** hydrogen sulfide, antibiotic resistance, resistance reversion, *Acinetobacter baumannii*, redox

## Abstract

The production of endogenous hydrogen sulfide (H_2_S) has been shown to confer antibiotic tolerance in all bacteria studied to date. Therefore, this mediator has been speculated to be a universal defense mechanism against antibiotics in bacteria. This is assuming that all bacteria produce endogenous H_2_S. In this study, we established that the pathogenic bacteria *Acinetobacter baumannii* does not produce endogenous H_2_S, giving us the opportunity to test the effect of exogenous H_2_S on antibiotic tolerance in a bacterium that does not produce it. By using a H_2_S-releasing compound to modulate the sulfide content in *A. baumannii*, we demonstrated that instead of conferring antibiotic tolerance, exogenous H_2_S sensitized *A. baumannii* to multiple antibiotic classes, and was able to revert acquired resistance to gentamicin. Exogenous H_2_S triggered a perturbation of redox and energy homeostasis that translated into hypersensitivity to antibiotic killing. We propose that H_2_S could be used as an antibiotic-potentiator and resistance-reversion agent in bacteria that do not produce it.

## Introduction

Antimicrobial resistance (AMR) is rising and poses a major public health threat (Sugden et al., 2016). The understanding of antibiotic modes of action (MOA), and bacteria mechanisms of resistance (MOR), is critically important in the efforts to develop alternative therapies. Over a decades ago, the formation of reactive oxygen species (ROS) has been proposed as a common effector mechanism in bacteria challenged with bactericidal antibiotics (Kohanski et al., 2007). Beyond the canonical drug-specific target-corruption MOA, the paradigm shifted toward system-level disruption of bacteria cellular homeostasis as a common mean of antibiotics-induced lethality (Kohanski et al., 2010). Accordingly, several studies reported system level MOR involving oxidative stress defenses. In 2011, a novel resistance mechanism mediated by hydrogen sulfide (H_2_S) was described for several pathogenic bacteria, including *Staphylococcus aureus*, *Pseudomonasaeruginosa*, *Escherichia coli*, and *Bacillus anthracis* (Shatalin et al., 2011). The model proposed that endogenously produced H_2_S reduces the cellular formation of ROS, by interfering with the Fenton reaction and by stimulating ROS-scavenging enzymes, thereby contributing to antibiotic tolerance. Genetic and chemical disruption of the H_2_S biosynthetic pathways resulted in exacerbated antibiotic sensitivity, suggesting that this pathway could be targeted to potentiate antibiotics or even revert resistance. Recently, however, exogenous H_2_S was shown to have cytotoxic effect on several microbes including *E. coli* (Wu et al., 2015; Fu et al., 2018).

Hydrogen sulfide is recognized as the third gasotransmitter in mammals, alongside nitric oxide (NO) and carbon monoxide (CO) and is implicated in diverse physiological processes (Wang, 2010). In mammals and some bacteria H_2_S is formed as a product of cysteine degradation either via cystathionine-synthase (CBS) and cystathionine-lyase (CSE), or via 3-mercaptopyruvate sulfur transferase (3MST). While some important pathogenic bacteria encode the H_2_S biosynthetic pathway, others do not (Luhachack and Nudler, 2014). For example, we show here that these genes are absent from the genome of *Acinetobacter baumannii*, a critically important antimicrobial resistance (AMR)-associated Gram-negative pathogen, while homologs can be found in the genome of *Klebsiella pneumoniae*. Antibiotic resistance mechanisms in *A. baumannii* include drug-modifying enzymes, efflux pump and reduced permeability (Asif et al., 2018). Here we investigate the effect of exogenous H_2_S on the antibiotic-susceptibility profile of non-H_2_S producing *A. baumannii*. We employed H_2_S-releasing agent and showed that exogenous H_2_S does not confer protection against ROS-inducing antibiotics. On the contrary, we showed that *A. baumannii* treated with H_2_S-releasing agent displayed hypersensitivity to different antibiotics with unrelated mechanisms of actions. Exogenous H_2_S treatment was also able to revert resistance against gentamicin-resistant *A. baumannii* isolates. We further showed that antibiotic-sensitization was mediated by system-level perturbation of redox homeostasis and energy metabolism. Our findings suggest that H_2_S cannot be regarded as a universal protective molecule against antibiotic insult. Instead, we propose that H_2_S-releasing compounds could be used in combination with antibiotics to sensitize AMR bacteria like *A. baumannii*.

## Materials and Methods

### Strains, Culture Conditions, and Chemicals

*Acinetobacter baumannii* Bouvet and Grimont (ATCC^®^ BAA-2093^TM^), clinical isolate #8879 (curtesy of Dr. Andrea Kwa and Dr. Lim Tze Peng, Singapore General Hospital (SGH), Singapore), and *Klebsiella pneumoniae* (Schroeter) Trevisan (ATCC^®^ 13883^TM^) were streaked on Luria-Bertani (LB)-agar plates. Single colonies were inoculated in LB broth at 37°C under agitation at 220 rpm. Overnight cultures were diluted 1:100 and grown to OD_600_ 0.6 to 0.8 for all assays. Colony forming units (CFUs) were determined by plating of 10-fold serial dilutions on LB-agar at 37°C overnight. Antibiotics and sodium hydrosulfide (NaHS) were purchased from Gold Biotechnology and Sigma-Aldrich, respectively. Solutions were prepared fresh in autoclaved and degassed double distilled water to 1M concentration.

### Time-Kill, ROS, Membrane Potential, Membrane Permeabilization, and ATP Level Determination

Time-kill curves were determined as follows: 5 × 10^6^ inoculum were treated with antibiotic, H_2_S donor or a combination of both at indicated concentrations. Samples were taken at various time points for CFU determination on agar plates. The effects of H_2_S on ROS accumulation and membrane depolarization were measured after 2 h of treatment in similar condition as time-kill assays. For ROS, 50 μL aliquots bacterial cultures were incubated for 30 min at 37°C under agitation at 220 rpm with 250 μM CellROX Green (Invitrogen) in a final volume of 250 μL of PBS. Bacteria treated with 20 μM of Menadione for 30 min were used as a positive control of ROS accumulation. For membrane depolarization, 50 μL aliquots bacterial cultures were incubated for 30 min at room temperature under agitation at 220 rpm with 150 μM DiOC2(3) + 0.5 μM EDTA in PBS in a final volume of 250 μL. We used treatment with 25 μM CCCP for 30 min as a positive control. Membrane permeability was determined using TO-PRO-3 Iodide dye (Invitrogen Molecular Probe). Briefly, 50 μL of treated culture was washed twice and resuspended in 0.8% NaCl with the dye as described by the manufacturer. Culture inoculum was used as a control of unpermeablized membrane and heat-killed bacteria were used as a positive control with over 98% of membrane permeabilization. Samples were processed by flow cytometry on a Attune NxT (ThermoFisher) flow cytometer. Fluorescence was measured for at least 100,000 events per sample. ATP level was determined using the BactiterGlo kit (Promega) according to manufacturer’s recommendations.

### Detection of H_2_S Production Using Lead-Acetate Paper and Monobromobimane (MBB) Derivatization Methods

Hydrogen sulfide production was detected in bacterial culture using lead-acetate paper strips (Johnson Test Papers). Briefly, a strip of paper was placed in bacterial tube in the air space above LB broth. Papers were collected and picture taken after 24 h of growth. For monobromobimane (MBB) derivatization, exponentially growing bacterial cultures were pelleted by centrifugation at 4,000 *g* for 10 min. A total of 20 μL of the supernatant was used in the MBB derivatization reaction. Briefly, 20 μL of the supernatant was added to 65 μL of 200 mM HEPES pH 8.2 and 1 μL of 100 mM MBB in acetonitrile for precisely 10 min before adding 5 μL of 50% (w/v) Trichloroacetic in water to stop the derivatization reaction. Samples were then centrifuged at 3,000 *g* for 5 min to pellet any residual debris and 80 μL of supernatant were transfer to autosampler vials and kept in 4°C in the dark before HPLC-MC analysis. HPLC-MS analysis followed the published reports with some modifications (Shen et al., 2015; Ditrói et al., 2019). Briefly, the derivatized samples were analyzed using Agilent 1290 ultrahigh pressure liquid chromatography system (Waldbronn, Germany) equipped with a 6550 QTOF mass detector managed by a MassHunter workstation. The column used for the separation was an Agilent rapid resolution HT Zorbax SB-C18 (2.1 × 50 mm, 1.8 mm; Agilent Technologies, Santa Clara, CA, United States). The gradient elution involved a mobile phase consisting of (A) 0.1% formic acid in water and (B) 0.1% formic acid in acetonitrile. The initial condition was set at 5% B. A 12 min linear gradient to 20% B was applied, followed by a 1 min gradient to 100% B which was held for 3 min, then returned to starting conditions over 0.1 min. Flow rate was set at 0.3 mL/min, and the auto-sampler was cooled at 4°C. A total of 5 μL of samples was injected. The electrospray ionization mass spectra were acquired in positive ion mode. Mass data were collected between m/z 100 and 1,000 at a rate of two scans per second. The electrospray ionization of the mass spectrometer was performed in positive ion mode with the following source parameters: drying gas temperature 250°C with a flow of 14 L/min, nebulizer gas pressure 40 psi, sheath gas temperature 350°C with a flow of 11 L/min, capillary voltage 3,500 V and nozzle voltage 500 V. Two reference masses were continuously infused to the system to allow constant mass correction during the run: m/z 121.0509 (C5H4N4) and m/z 922.0098 (C18H18O6N3P3F24). Raw spectrometric data were analyzed by MassHunter Qualitative and Quantitative Analysis software (Agilent Technologies, United States).

### MRR Detection and Measurements

All ^1^H magnetic resonance relaxometry (MRR) measurements were performed in bench top MRR spectrometer which consists of a portable permanent magnet (Metrolab Instruments, Plan-les-Ouates, Switzerland) with *B*_0_ = 0.5T and a bench-top type NMR console (Kea Magritek, Wellington, New Zealand) at the resonance frequency of 21.65 MHz inside the magnet. A single resonance proton MRR probe with detection micro coil of 900-μm inner diameter was used for accommodating the MRR samples into the micro capillary tubes (o.d.: 1,500 μm, i.d.: 950 μm) (22-260-950, Fisherbrand, Waltham, MA, United States). In MRR probe, the electronic parts and coil were mounted on the single printed circuit board. Bacterial cultures aliquots were centrifuged at 4,000 *g* for 10 min. The pellet was resuspended in 50 μL of PBS and loaded into 4 mm length (detection range of coil) of micro capillary tube. The capillary tube was sealed by crystoseal and mounted into the coil. All the experiments were performed at 26.3°C inside the magnet which is maintained by a temperature controller (RS component, United Kingdom). Proton transverse relaxation rates T_2_ were measured by standard Carr-Purcell-Meiboom-Gill (CPMG) pulse program. We maintained the transmitter power output at 12.5 mW for a single 90° pulse of pulse length 16 μs for all the T_2_ measurements. The CPMG train of pulses with inter echo time of 60 μs with 4,000 echoes were used for all experiments. A recycle delay of 2 s, which is enough to allow all the spins to return to thermal equilibrium, was used. 24 scans were performed for all experiments for signal averaging.

### Sample Preparation for Proteomic Analysis

The bacterial pellet was resuspended in 6× volume of 8 M urea containing 1 mM sodium orthovanadate and homogenized using a sonicator pulse for 3 min at 27% amplitude and 1 s on, 4 s off pulse time. The lysate was spun at 16,000 *g* at 4°C for 30 min to pellet the insoluble fraction and the lysate was transferred into a new tube. After carrying out a BCA Assay (Thermo), 100 μg of protein was reduced with 10 mM DTT at 56°C for 1 h and followed by alkylation using 100 mM IAA for 1 h in the dark. This solution was diluted to 1 M urea and digested with 2 μg Trypsin (Thermo) overnight at ambient temperature. The resulting peptides were acidified using formic acid and desalted using Pierce desalting columns as per manufacturer’s instructions. These peptides were reconstituted in triethylammonium bicarbonate (TEAB) and labeled using tandem-mass-tag TMT labels (Thermo) as per manufacturer’s instructions. The labels for the peptides for the first run were as follows 127N-BioRep1-Untreated, 127C-BioRep1-NAHS_Colistin_Treated, 128N – BioRep1-Colistin_Treated, 128C – BioRep1-NAHS_Treated, 129C-BioRep2-Untreated, 130N-BioRep2-NAHS_Colistin_Treated, 130C – BioRep2-Colistin_Treated, and 131 – BioRep2-NAHS_Treated. The labels for the second run were as follows 129C-BioRep3-Untreated, 130N-BioRep3-NAHS_Colistin_Treated, 130C – BioRep3-Colistin_Treated, and 131 – BioRep3-NAHS_Treated. The labeled peptides were combined, dried, and reconstituted in 0.1% FA. The peptides were then further fractionated using high-pH fractionation columns (Pierce) as per manufacturer’s instructions into eight fractions.

### Mass Spectrometry

Peptides were separated by reverse phase HPLC (Thermo Easy nLC1000) using a self-packed 20 cm Picofrit column (New England Objective) over a 90 min gradient before nano-electrospray using an Orbitrap Fusion Tribrid mass spectrometer (Thermo Scientific). The mass spectrometer was operated in a data-dependent mode. The parameters for the full scan MS were: resolution of 60,000 across 350–1,500 m/z, AGC 1e^3 and maximum injection time (IT) 50 ms. The full MS scan was followed by MS/MS for a total cycle time of 3 s with an NCE of 34 and dynamic exclusion of 30 s and maximum IT of 300 ms at a resolution of 60,000. Raw mass spectral data files (.raw) were searched using Proteome Discoverer (Thermo Fisher) and MASCOT (Perkins et al., 1999). The reference proteome for *A. baumannii* on Uniprot^1^ was searched using the following parameters were: 10 ppm mass tolerance for precursor ions; 0.08 Da for fragment ion mass tolerance; two missed cleavages of trypsin; fixed modification was carbamidomethylation of cysteine; variable modifications were methionine oxidation, phosphorylation (serine and tyrosine) and TMT-10plex label. Only peptides with a Mascot score greater than or equal to 10 and an isolation interference less than or equal to 30 were included in the data analysis. The data was subsequently analyzed using the MSStatsTMT R package (Huang et al., 2019). The functional enrichment was carried out using TOPGO (Alexa and Rahnenführer, 2019) and Blast2GO (Götz et al., 2008).

## Results

### *A. baumannii* Does Not Produce H_2_S and Exogenous H_2_S Sensitizes It to Different Antibiotics

**FIGURE 1:**
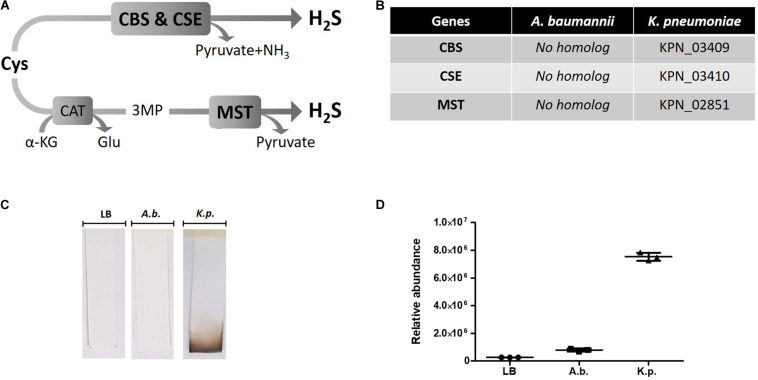
H_2_S pathway and detection in *A. baumannii* and *K. pneumoniae*. **(A)** H_2_S biosynthetic pathway. Cysteine (Cys), cystathionine-beta-synthase (CBS), cystathionine-gamma-lyase (CSE), alpha-ketoglutarate (α-KG), glutamate (Glu), 3MP (3-mercaptopyruvate), cysteine aminotransferase (CAT), 3-mercaptopyruvate sulfurtransferase (MST). **(B)** H_2_S pathway gene orthologs. Genome search identified conserved CBS, CSE and MST in *K. pneumoniae* but not in *A. baumannii*. **(C)** Detection of H_2_S production by oxidation of lead-acetate paper strip. **(D)** Quantification of H_2_S by monobromobimane derivatization and liquid chromatography. Three independent biological replicates are shown with mean and standard deviation. Statistical analysis was conducted using unpaired *t* test with Welsh correction, two-tailed *P* value with *p* < 0.0001 (***).

**FIGURE 2:**
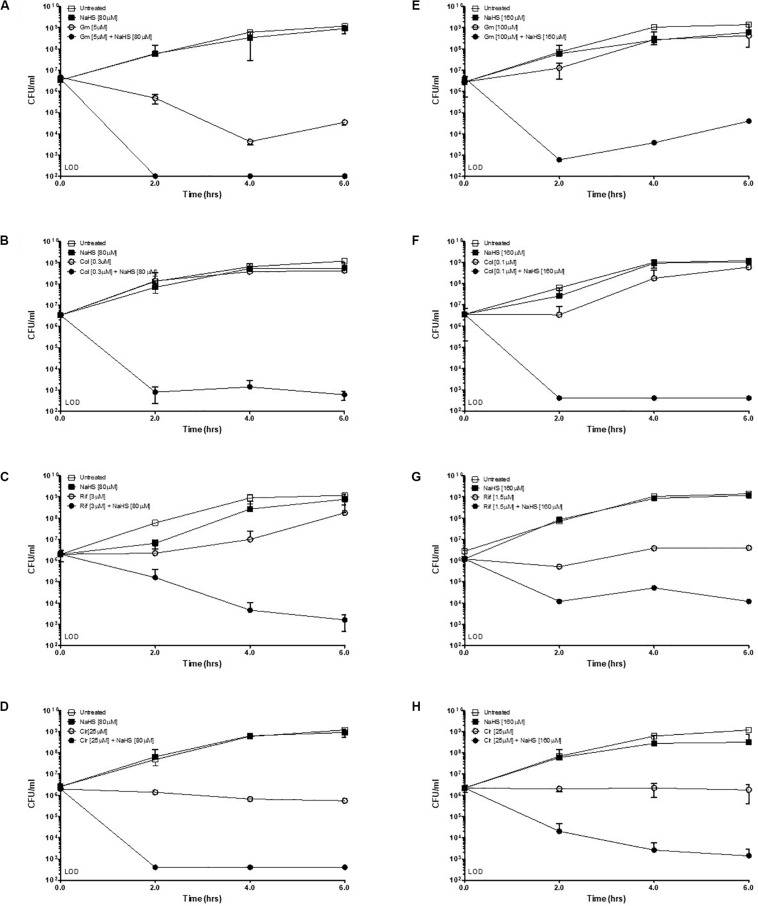
Exogenous H_2_S sensitizes *A. baumannii* to killing by antibiotics. Time-kill experiments were conducted with two different isolates of *A. baumannii*, ATCC #2093 **(A–D)** and clinical isolate #8879 **(E–H)**. Briefly, 10^6^ bacteria were treated with either a sub-lethal concentration of antibiotic alone or combined with the H_2_S-releasing molecule NaHS (sodium hydrosulfide) at given concentrations. Survival was determined by colony enumeration on agar plates at indicated time points. Gm, Gentamicin; Col, Colistin; Rif, Rifampicin; Clr, Clarithromycin. Experiments were carried in two independent biological replicates. Representative results are shown.

Hydrogen sulfide has been suggested to confer universal protection against different antibiotics in H_2_S-producing bacteria (Shatalin et al., 2011). We investigated the effect of exogenous H_2_S on *A. baumannii*, a critically important AMR bacteria that does not carry the genes coding for the H_2_S biosynthetic pathway, i.e., CBS, CSE or MST (Figure 1A). A BLAST search of the genome of *A. baumannii* ATCC^®^ BAA-2093^TM^ did not reveal any orthologs. *Klebsiella pneumoniae*, another Gram-negative bacterium, carried all three genes (Figure 1B). We confirmed that *A. baumannii* does not produce H_2_S using both the lead-acetate paper strip and the monobromobimane H_2_S detection methods (Betty et al., 2007; Shen et al., 2015) (Figures 1C,D). Conversely H_2_S production was detected in *K. pneunoniae* (Figures 1B,C). We next made use of sodium hydrosulfide (NaHS), a compound releasing H_2_S instantly in aqueous solution (Supplementary Figure 1), to probe the effect of exogenous H_2_S on *A. baumannii* physiology and antibiotic tolerance. To determine if H_2_S affects antibiotic sensitivity in *A. baumannii*, we conducted antibiotic time-kill experiments by CFU determination overtime in the absence or presence of NaHS. Surprisingly, these experiments revealed that exogenous H_2_S did not confer protection against antibiotics in *A. baumannii*, but in fact potentiated the killing effects of mechanistically unrelated antibiotics including gentamycin, colistin, rifampicin, and clarithromycin (Figure 2). When cultures of antibiotic-sensitive *A. baumannii* were co-treated with H_2_S-releasing compounds and antibiotics, the bactericidal activity of these antibiotics was several orders of magnitude greater than the antibiotics alone. For example, while gentamycin alone killed only 90% of the inoculum in 2 h, addition of NaHS resulted in complete eradication (Figure 2A). A similar trend was observed with colistin (Figure 2B). Interestingly, the antibiotics rifampicin and clarithromycin that were bacteriostatic at best when tested alone were able to induce a dramatic 99.9% reduction in viability in the presence of NaHS (Figures 2C,D). Similar finding was observed in a different *A. baumannii* clinical isolate (Figures 2F–H). We observed that this second isolate is resistant to gentamicin up to 100 μM. Despite acquired resistance to gentamycin, co-treatment with H_2_S-releasing agent and gentamicin translated into bactericidal activities, achieved over 3-log of CFU in 2 h (Figure 2E). Contrary to what was reported in *E. coli*, *S. aureus*, *P. aeruginosa*, and *B. anthracis*, H_2_S did not protect *A. baumannii* against antibiotic insult, but in fact hypersensitized susceptible isolates to killing by different class of antibiotics and reverted resistance in a drug-resistant isolate of *A. baumannii*. This suggests that the mechanism of H_2_S-induced antibiotic sensitization is independent of antibiotic mechanism of action. We hypothesized that H_2_S exerts its antibiotic-sensitization effect by compromising bacterial cellular redox homeostasis and reducing the ability of the bacteria to deal with antibiotic-mediated lethal mechanisms, including oxidative stress.

### H_2_S Induces Perturbations of Redox and Energy Homeostasis

**FIGURE 3:**
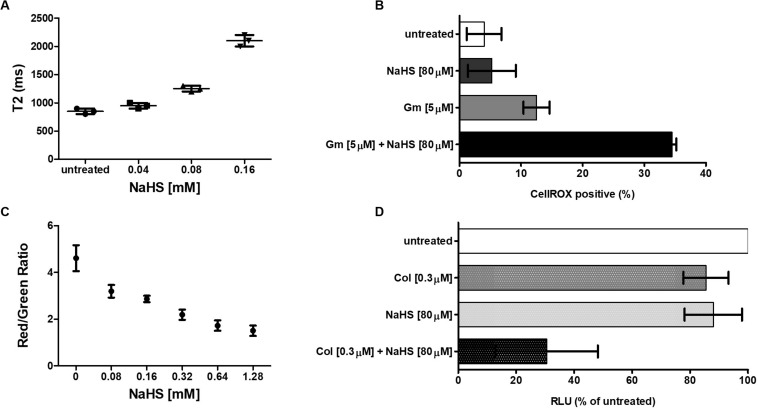
H_2_S induces perturbation of redox and energy homeostasis. **(A)** Effect of H_2_S on iron oxidation status. A total of 10^6^ bacteria were treated with increasing concentration of NaHS and T2 values were measured by MRR after 2 h of treatment. Cfu were enumerated on agar plate for normalization. **(B)** Effect of H_2_S on oxidative stress level. A total of 10^6^ bacteria were exposed to either 80 μM of NaHS, 5 μM of Gentamicin, or a combination of both. Bacteria were collected after 4 h of treatment, washed, and resuspended in PBS containing the CellROX dye. The percentage of CellROX positive bacteria was determined by flow cytometry. **(C)** Effect of H_2_S on the membrane potential. A total of 10^6^ bacteria were exposed to increasing concentration of NaHS for 2 h after which the membrane potential was measured by the DiOC2 (3,3’-diethyloxacarbocyanine iodide) fluorescence assay. **(D)** Effect of H_2_S on the intracellular ATP level. A total of 10^6^ bacteria were exposed to either 80 μM of NaHS, 0.3 μM of Colistin, or a combination of both for 4 h, after which the ATP content was measured by ATP-dependent luciferin/luciferase assay. RLU is reported as a percentage of untreated cells. All experiments were performed twice in independent biological replicates. Means and standard deviations are shown. Statistical analysis was conducted using unpaired t test with two-tailed *P* value as follow: *p < 0.05 and **p < 0.005.

Hydrogen sulfide cytotoxic effects, notably caused by its interaction with iron-containing protein, are well documented (Reiffenstein et al., 1992). Iron-containing proteins play an important role in bacterial metabolism and respiration and are also involved redox sensing (Green and Paget, 2004). We used MRR to determine the effect of H_2_S on intracellular iron as a proxy for redox status of in *A. baumannii* following treatment with NaHS. T2 MRR, i.e., the measure of protons relaxation, is most frequently used for iron quantification (Dusek et al., 2013). The transformation of ferrous Fe^2+^ into ferric Fe^3+^ induces measurable changes in the magnetic resonance relaxation of nearby protons that can be measured by T2 MRR. We treated cultures of *A. baumannii* with increasing concentration of NaHS alone, or in combination with a fixed concentration of antibiotic. T2 values were measured and normalized per CFU. We showed that NaHS alone induces a dose-dependent increase in T2 values, reflecting an increase in Fe^2+^/Fe^3+^ ratio (Figure 3A). When combined with colistin, T2 values increase was significantly higher (Supplementary Figure 2) than with either colistin or NaHS alone. Considering the role played by Fe^2+^ in ROS generated via Fenton chemistry (Winterbourn, 1995), we next determined how H_2_S influence the oxidation of the CellROX fluorescent probe, commonly used to measure oxidative stress status in cells. Our results showed that treatment with NaHS alone resulted in a dose-dependent increase of CellROX-positive bacteria (Supplementary Figure 3). Given that oxidative stress and redox disbalance has been implicated in antibiotics lethality (Kohanski et al., 2007; Dwyer et al., 2014), we hypothesized that H_2_S potentiates antibiotic-induced oxidative-stress. To test that hypothesis, we quantify CellROX positive cells following 2-h treatment with gentamicin. The combination of NaHS and gentamicin resulted in significant increase of CellROX positive bacteria when compared to NaHS or gentamicin alone. H_2_S is also known to inhibit respiratory cytochromes. The activity of the respiratory chain is involved in the maintenance of the membrane potential and the generation of ATP. Both are critical for bacteria survival. If H_2_S inhibits cytochromes of the respiratory chain, it should interfere with the membrane potential. We used the 3,3-diethyloxacarbocyanine iodide (DiOC2) probe to measure membrane potential (Novo et al., 2000). NaHS induced a significant depolarization of the membrane as illustrated by a dose-dependent drop in the red/green ratio of the probe (Figure 3C). Since ATP synthesis requires membrane polarization, we quantified ATP levels in bacteria treated with H2S. ATP levels were markedly reduced following treatment with NaHS (Figure 3D). We conducted a proteomic analysis on *A. baumannii* treated with either NaHS or Colistin, alone or in combination (Supplementary Figure 5). Results showed that ATP synthase and iron-sulfur cluster proteins were upregulated following treatment with combination of NaHS and Colistin. Both the terminal enzyme of the respiratory chain and iron sulfur cluster proteins are associated with pro-oxidative metabolism. Conversely, proteins associated with stress response and oxidative stress defenses, as well as central carbon metabolism, were downregulated (Table 1 and Supplementary Table 1). The glutathione biosynthetic pathway, which contributes to maintenance of the proper oxidation state of protein thiols and protections against oxidative stressors, was markedly affected. The expression of superoxide dismutase protein, a major oxidant detoxifying enzyme, was significantly reduced. Finally, protein grouped under the generic ontological term of “stress protein” have been shown to protect cells from damaging effects of ROS and mediate antibiotic resistance (Elhosseiny et al., 2015; Gebhardt et al., 2015). The treatment with a combination of NaHS and colistin induced a marked decrease in each of these protein families, rendering these defense systems ineffective. Taken together, the results presented above suggest that H_2_S act on *A. baumannii* at system level and induces disruption of redox and energy homeostasis.

**TABLE 1:** Proteomic analysis.

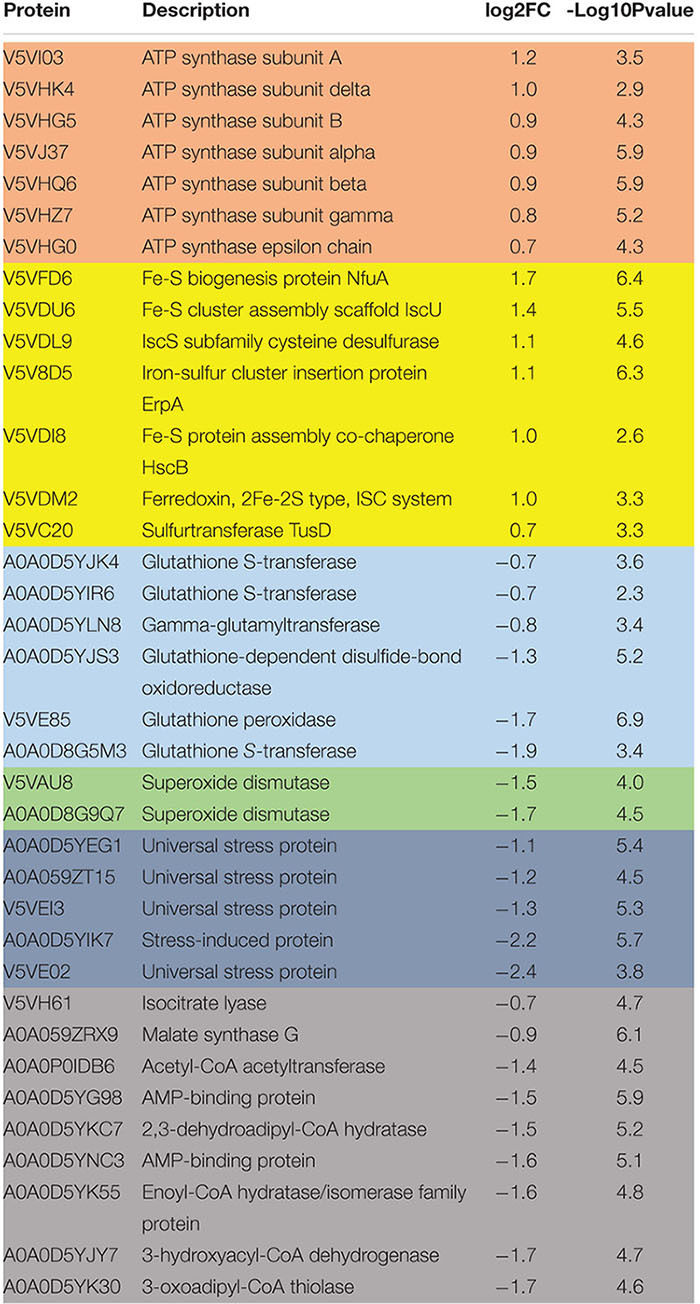

*Curated list of differentially expressed proteins in *A. baumannii* following treatment with both NAHS and Colistin relative to the untreated control. Protein numbers are reported as per the Uniprot database. Fold change (FC) values are aggregate ratios of treated samples to untreated control. *p*-value refers to the probability of the null hypothesis being valid after adjusting for multiple comparisons. ATP synthase proteins (pink) and iron-sulfur cluster proteins (yellow) show increased protein expression. Oxidative stress response proteins which are glutathione transferases (light blue) and superoxide dismutases (green) show a decrease in protein expression. Universal stress response (purple) and glycolysis proteins (gray) also show a decrease in protein expression. These proteins are depicted by blue dots in the volcano plot in Supplementary Figure 6. They are additionally represented in the STRING analysis as well Supplementary Figures 7 and 8.*

## Discussion

Antibiotics exert their bactericidal effect by interfering with several essential pathways downstream of their target interactions (Kohanski et al., 2010). For example, corruption of redox homeostasis is a common mode of action of several antibiotics. Conversely, bacteria use system-level mechanisms that contribute to antibiotic tolerance. H_2_S has been shown to render multiple bacterial species tolerant to oxidative stress and various classes of antibiotics (Shatalin et al., 2011). H_2_S was proposed as a universal defense mechanism against antibiotic insults. That is assuming that all bacteria produce H_2_S. Here we show that *A. baumannii*, an important AMR pathogen, does not encode the H_2_S biosynthetic pathway and does produce H_2_S. We used an H_2_S-releasing molecule to modulate the intracellular H_2_S content of *A. baumannii* and asked how H_2_S influences its antibiotic-susceptibility profile. We showed that exogenous H_2_S sensitizes *A. baumannii* to killing by mechanistically unrelated antibiotics and reverted resistance to gentamycin in a clinical isolate. To our knowledge, this is the first report of H_2_S-induced antibiotic sensitization and resistance reversion. Phenotypic analysis revealed that H_2_S affects the bacterial Fe^2+^/Fe^3+^ balance toward Fe^2+^, possibly via direct reduction of Fe^3+^ cations by sulfide. Treatment with H_2_S donor also increased CellROX positive cells, reflecting a pro-oxidant redox status. Energy metabolism was affected as indicated by a reduced membrane potential and a decrease in ATP production. Membrane permeability remained unchanged (Supplementary Figure 4). Proteomic analysis supported these phenotypic results with up-regulation of iron-sulfur cluster proteins and ATP synthase subunits and down-regulation of proteins associated with oxidative stress responses and central carbon metabolism. Our results suggest that H_2_S interferes with redox and energy homeostasis in *A. baumannii* leading to sensitization to killing by mechanistically unrelated antibiotics.

Beneficial as well as detrimental effects of H_2_S in animals and plants have been widely reported. Interestingly, both effects are related to either protection against, or exacerbation of, oxidative damages and energy metabolism (Tanaka et al., 1968; Beauchamp et al., 1984; Eghbal et al., 2004; Kimura and Kimura, 2004; Attene-Ramos et al., 2007; Joyner-Matos et al., 2010; Kimura et al., 2010). One of the main differences between these studies was the concentrations range of H_2_S or H_2_S-donors that have been used. Generally, low concentrations (micromolar) of H_2_S are regarded as cytoprotective while high concentrations (millimolar) are cytotoxic (Ritter, 2010). Several studies have also established that H_2_S can either be cytoprotective or cytotoxic to microbes (Shatalin et al., 2011; Wu et al., 2015; Ooi and Tan, 2016; Fu et al., 2018). Shatalin et al. (2011) showed that H_2_S is produced endogenously by orthologs of CBS, CSE, or MST in *B. anthracis*, *P. aeruginosa*, *S. aureus*, and *E. coli*. In these bacteria, the H_2_S-mediated protective mechanism was two-fold: suppression of oxidant generated by the Fenton reaction and stimulation of antioxidant enzymes (Shatalin et al., 2011). However, Weikum et al. (2018) recently showed that, in *S. aureus*, H2S-mediated protection was limited to aminoglycosides, while it actually exacerbated the killing by other antibiotics like quinolones. More importantly, H2S-induced tolerance to gentamicin was not due to oxidative stress reduction, but rather caused by the decrease in gentamicin uptake. We authors argued that H2S cannot be regarded as a general defense mechanism against antibiotics. Conversely, H_2_S cytotoxic effects involved the generation of oxidative stress and the suppression of antioxidant defenses. H_2_S antifungal effects on *Aspergillus niger* and *Penicillium italicum* were mediated by reduction in superoxide dismutase (SOD) and catalase (CAT) activities, and increased ROS formation (Fu et al., 2014). Wu et al. (2015) revealed that H_2_S promoted H_2_O_2_ killing by inactivating the heme-containing enzyme CAT in *Shewanella oneidensis* and Fu et al. (2018) showed that H_2_S triggers oxidative damages in *E. coli*.

In line with these findings, our results indicate that in *A. baumannii*, H_2_S triggers a pro-oxidative redox disbalance that exacerbate antibiotic-mediated killing. *A. baumannii* is a critically important AMR pathogen for which therapeutic options are increasingly limited with isolates now displaying resistance to last resort drug including colistin. H2S-releasing molecules have been developed for a wide range of clinical applications (Gemici et al., 2015; Wen et al., 2018). Their ability to increase H2S concentration in several body compartments, along with their safety profile, have been reported (Toombs et al., 2010; Wallace, 2015; Wallace et al., 2020). We propose that H_2_S-releasing compounds could be used as antibiotic-potentiators and resistance-reversion agents in *A. baumannii* and in bacteria that do not produce it.

## Data Availability Statement

The datasets generated for this study can be found in the https://chorusproject.org/pages/authentication.html#/login; accession number PXD020384.

## Author Contributions

WM conceived the study. SN, KO, JJL, JC, SS, LT, LC, HL, PH, AS, and JL conducted the experiments. WM, JH, SN, KO, AS, and SS analyzed the data. WM wrote the manuscript. All authors contributed to the article and approved the submitted version.

## Conflict of Interest

The authors declare that the research was conducted in the absence of any commercial or financial relationships that could be construed as a potential conflict of interest.

## Footnotes

1 https://www.uniprot.org/proteomes/UP000032746
