# Supplementary material for: Hydrogen Sulfide Sensitizes Acinetobacter baumannii to Killing by Antibiotics
Source: Front Microbiol. 2020 Aug 7;11:1875. doi: 10.3389/fmicb.2020.01875 (PMC7427342; doi:10.3389/fmicb.2020.01875)
Supplement: Supplementary file 2 [file Table_2.docx]

**Supplementary Material**

Hydrogen sulfide sensitizes *Acinetobacter baumannii* to killing by antibiotics.

**Authors:** Say Yong NG^1#^, Kai Xun ONG^1,2#^, Smitha Thamarath SURENDRAN^1,4^, Ameya SINHA^1,6,7^, Joey Jia Hui LAI^1,3^, Jacqueline CHEN^1^, Jiaqi LIANG^1,7^, Leona Kwan Sing TAY^1^, Cui Liang^1^, Hooi Lin LOO^1^, Peiying HO^1^, Jongyoon HAN^1,4,5^, Wilfried MOREIRA^1^*

**Table of Content**

Supplementary Figure 1. Quantification of H_2_S released as a function of NaHS concentration

Supplementary Figure 2. Effect of H_2_S and colistin on iron oxidation status

Supplementary Figure 3. Effect of H_2_S on oxidative stress level.

Supplementary Figure 4. Effect of H_2_S on membrane permeability.

Supplementary Figure 5. Schematic depicting the workflow for proteomics experiments

Supplementary Figure 6. Volcano plot of cumulative proteomic data.

Supplementary Figure 7. STRING analysis of up-regulated proteins

Supplementary Figure 8. STRING analysis of down-regulated proteins

Supplementary Excel File. Complete Proteomic Dataset (separate file)

**Supplementary Figures**

**
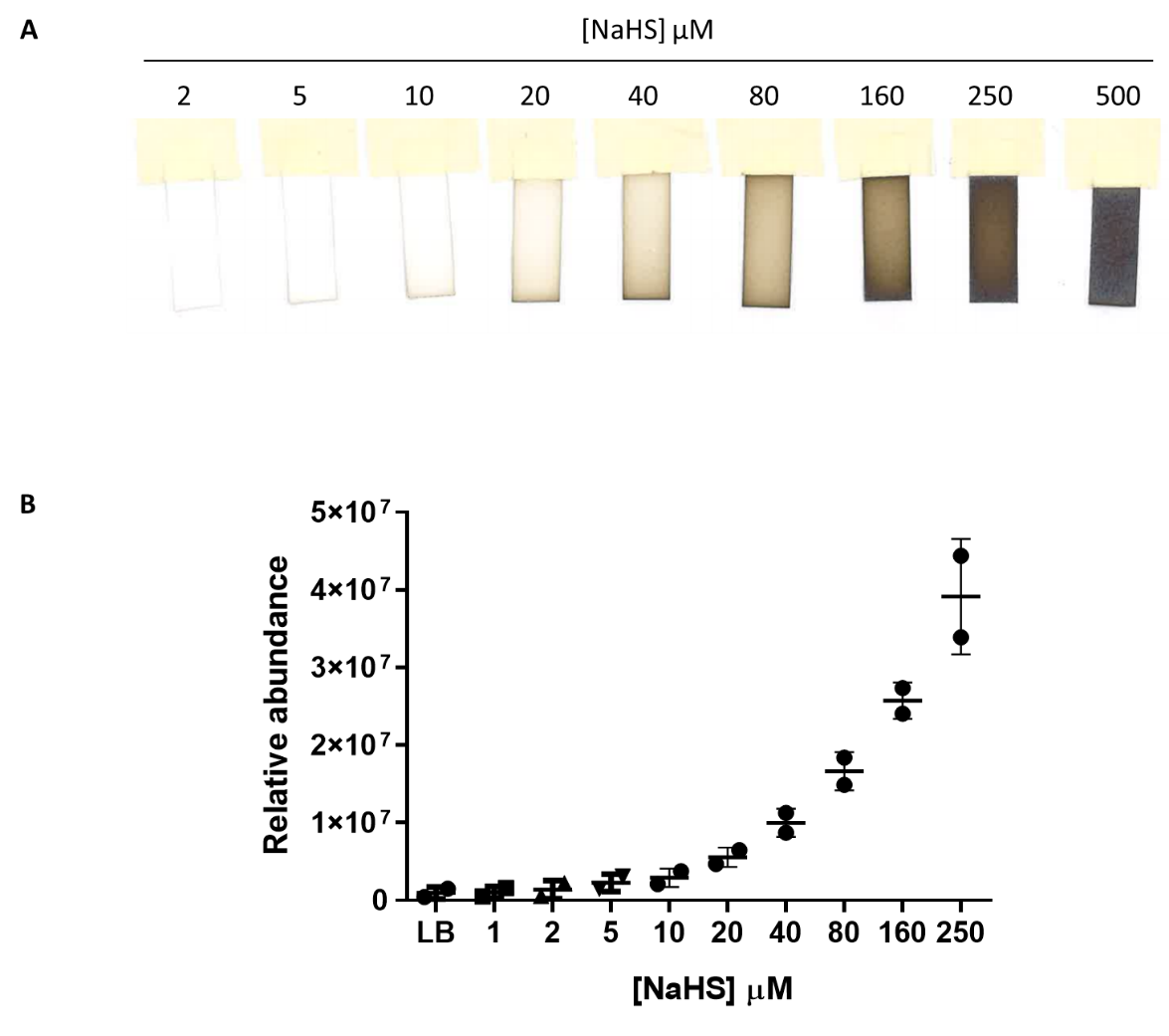
**

**Supplementary Figure 1. Effect of H_2_S and colistin on iron oxidation status**. **A**. Detection of H_2_S released by NaHS in air space of bacterial culture tube by oxidation of lead-acetate paper strip **B**. Quantification of H_2_S released by NaHS in solution via monobromobimane derivatization and LC-MS.

**Supplementary Figure 2. Effect of H_2_S and colistin on iron oxidation status**. 10^6^ bacteria were treated with increasing concentration of colistin in presence or absence of 80 µM of NaHS. T2 values were measured by MRR after 4 hours of treatment. Cfu were enumerated on agar plate for normalization.

**Supplementary Figure 3. Effect of H_2_S on oxidative stress level**. 10^6^ bacteria were exposed to increasing concentration of NaHS. Bacteria were collected after 4 hours of treatment, washed, and resuspended in PBS containing the CellROX dye. The percentage of CellROX positive bacteria was determined by flow cytometry.

**Supplementary Figure 4. Effect of H_2_S on membrane permeability**. 10^6^ bacteria were exposed to increasing concentration of NaHS for 4 hours after which the percentage of permeabilized bacteria was quantified using TO-PRO-3 fluorescent dye**.**


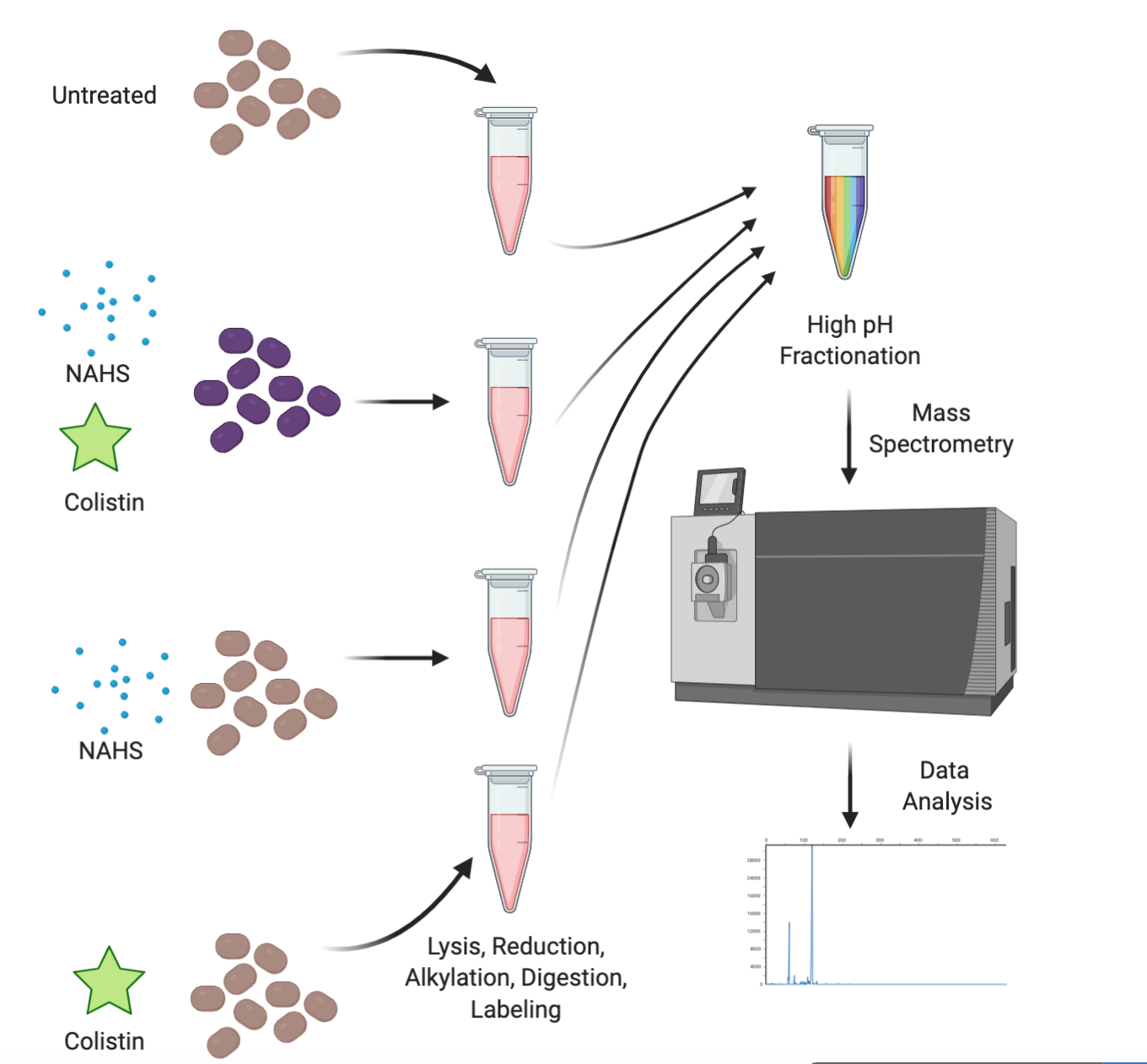


**Supplementary Figure 5. Schematic depicting the workflow for proteomics experiments** Log-growing *A. baumanii* cultures were treated in triplicates in the following conditions: 1. Untreated Control, 2. 80 uM of NAHS and 0.3 uM of Colisitin, 80 uM of NAHS alone, 4. 0.3 uM of Colisitin alone. After four hours, the cell pellets were lysed in a 6M Urea buffer, followed by reduction, alkylation, trypsin digestion, and were labelled with TMT-10plex labels as described in methods section XX. Labelled peptides were then combined and subjected to high-pH fractionation and analyzed on an Orbitrap Fusion Mass Spectrometer. The data was subsequently analysed using Thermo PD software and MSTMTStats to infer protein levels in each of these conditions and is provided as Supplementary Excel File – Complete Proteomic Dataset (separate file).

**Supplementary Figure 6.** **Volcano plot of cumulative proteomic data.** Volcano plot depicts protein expression changes relative to the untreated control as log_2_(foldchange) on the x-axis and significance as (-)log_10_(p.value) on the y-axis. Dotted lines at x=1.3 and y=$\pm$0.7 represent cut-offs for significant fold-changes for subsequent analysis. Green (●) and orange points (●) represent values from the Colistin alone and NAHS alone treated cultures respectively at sub-inhibitory concentrations. All of these points either fell below our thresholds for significant fold changes indicating no detectable changes from untreated control cultures. The other colors represent proteins expression changes from the culture treated with both NAHS and Colistin. Proteins represented by grey (○) points do not make thresholds for significant fold changes. Proteins represented by red points (○) are significantly different and utilized for GO and STRING analysis. Proteins in blue points (●) are a subset of significantly different proteins that drew our interest and are listed in Table 1.


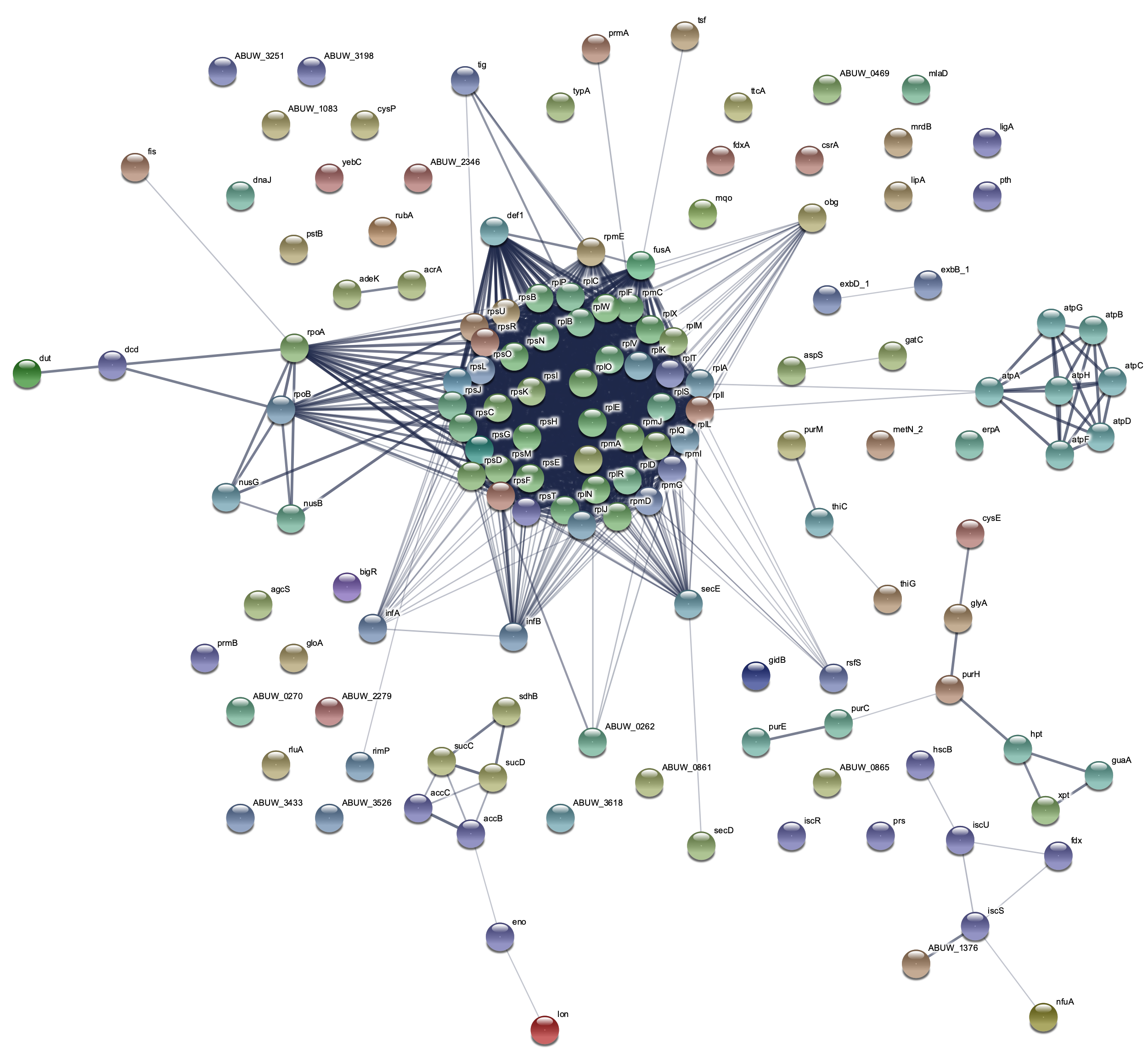


**Supplementary Figure 7. STRING analysis of up-regulated proteins.** Up-regulated proteins as defined by a cutoff of 0.7>log_2_(foldchange) and p-value<0.05 were plugged into the STRING-database (<https://string-db.org/>) to infer relevant protein-protein interactions in cells treated with both NAHS/Colistin. Strikingly, several ribosomal proteins were enriched as part of the central cluster. The other three well defined clusters were the ATP synthase complex, iron-sulfur proteins, and citrate acid cycle proteins. The STRING analysis parameters were interactions inferred from databases or experimental validation only at high confidence.


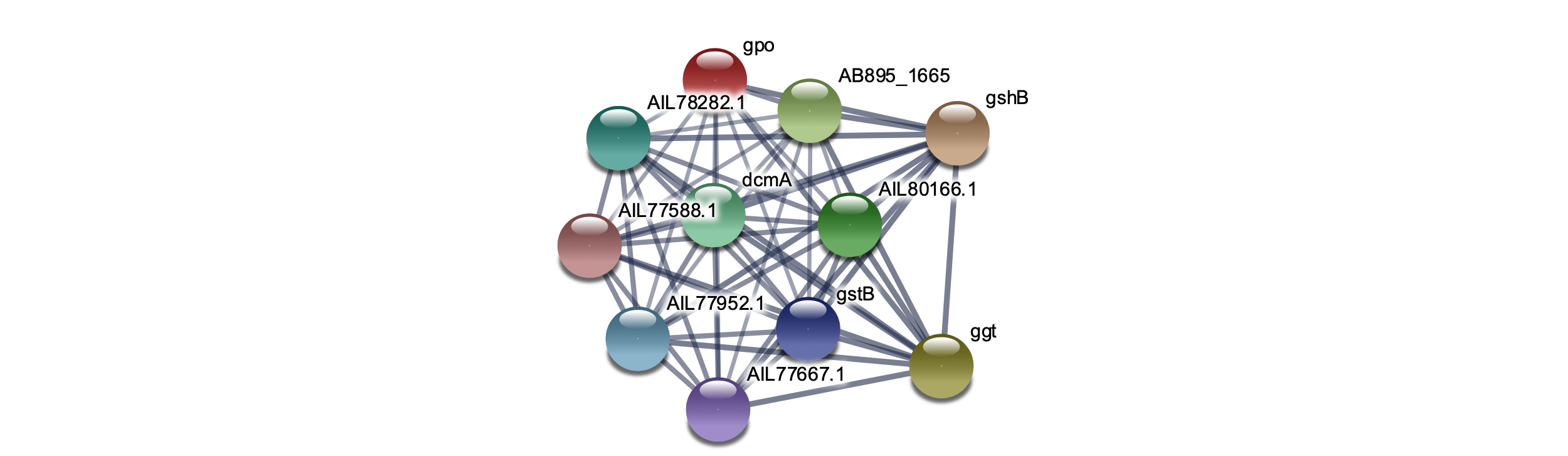

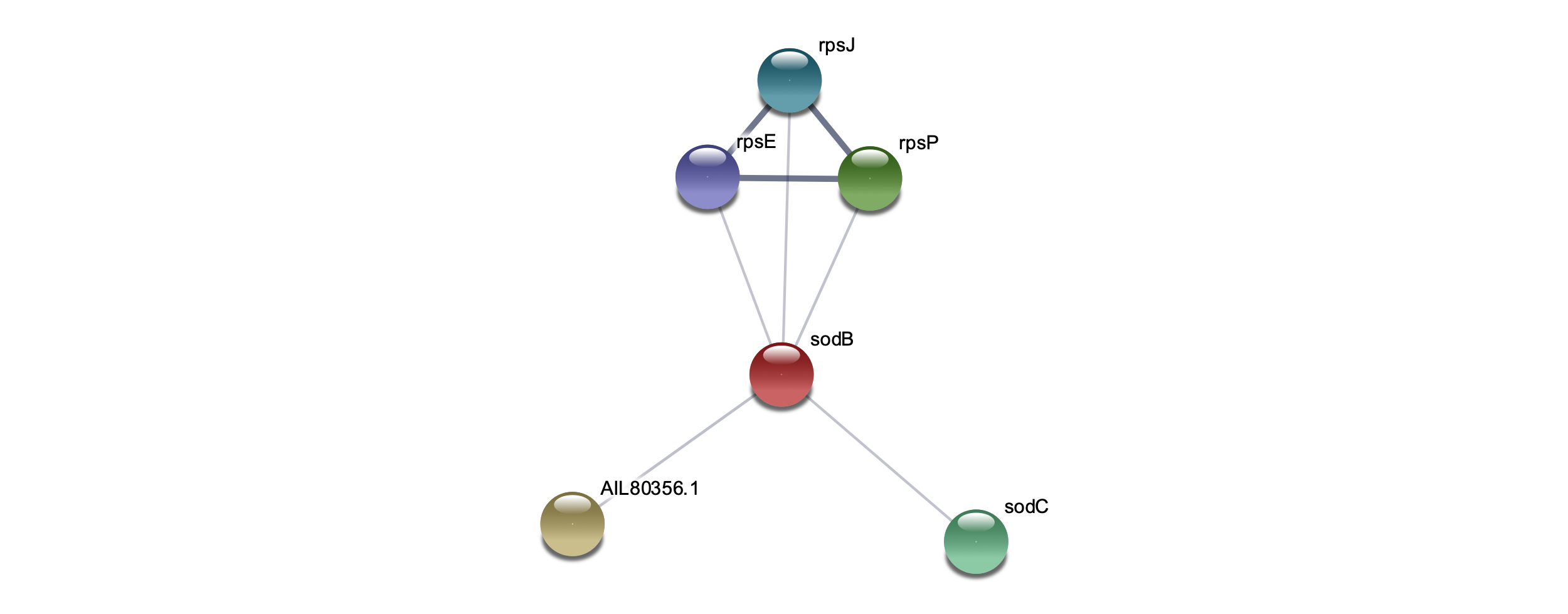

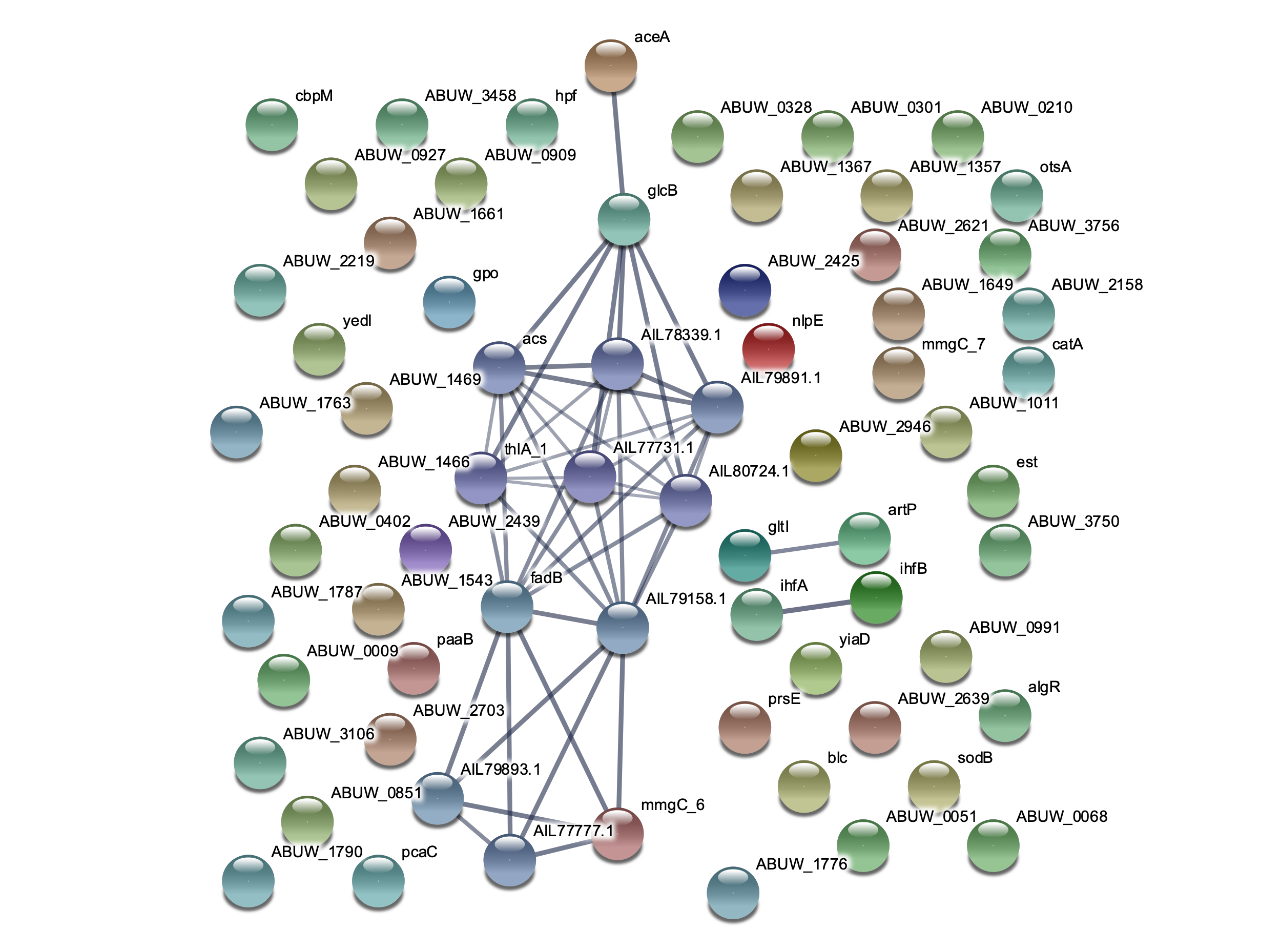


**Supplementary Figure 7. STRING analysis of up-regulated proteins**. Down-regulated proteins as defined by a cutoff of -0.7>log_2_(foldchange) and p-value<0.05 were plugged into the string-database (<https://string-db.org/>) to infer relevant protein-protein interactions. Strikingly, several ribosomal proteins were enriched as part of the central cluster. Oxidative stress proteins which include a cluster of glutathione transferases and superoxide dismutases were depleted in NAHS/Colistin treated cells. The other major cluster that was evident were glycolysis-related proteins. The STRING analysis parameters were interactions inferred from databases or experimental validation only at high confidence.

**Data Availability**

<https://chorusproject.org/pages/dashboard.html#/projects/my/1654/>
